# Diverse Expression of Antimicrobial Activities Against Bacterial Vaginosis and Urinary Tract Infection Pathogens by Cervicovaginal Microbiota Strains of *Lactobacillus gasseri* and *Lactobacillus crispatus*

**DOI:** 10.3389/fmicb.2019.02900

**Published:** 2019-12-20

**Authors:** Fabrice Atassi, Diane L. Pho Viet Ahn, Vanessa Lievin-Le Moal

**Affiliations:** ^1^ISNERM UMR-S 1166, Sorbonne University, Paris, France; ^2^INSERM, UMR-S 1166, CHU Pitié-Salpêtrière, Faculty of Medicine, Paris, France; ^3^INSERM UMR-S 996, University of Paris-Sud, Orsay, France; ^4^INSERM UMR-S 996, Paris-Saclay University, Saint-Aubin, France; ^5^INSERM, UMR-S 996, Clamart, France

**Keywords:** cervicovaginal microbiota, *Lactobacillus gasseri*, *Lactobacillus crispatus*, bacterial vaginosis, urinary tract infections, antimicrobial

## Abstract

We aimed to analyze the strain-by-strain expression of a large panel of antimicrobial activities counteracting the virulence mechanisms of bacterial vaginosis-associated *Prevotella bivia* CI-1 and *Gardnerella vaginalis* 594, pyelonephritis-associated *Escherichia coli* CFT073, and recurrent cystitis- and preterm labor-associated IH11128 *E. coli* by *Lactobacillus gasseri* and *Lactobacillus crispatus* clinical strains, and *L. gasseri* ATCC 9857 and KS 120.1, and *L. crispatus* CTV-05 strains isolated from the cervicovaginal microbiota of healthy women. All *L. gasseri* and *L. crispatus* strains exerted antimicrobial activity by secreted lactic acid, which killed the microbial pathogens by direct contact. Potent bactericidal activity was exerted by a very limited number of resident *L. gasseri* and *L. crispatus* strains showing the specific ability to a strain to produce and release antibiotic-like compounds. These compounds eradicated the microbial pathogens pre-associated with the surface of cervix epithelial cells, providing efficient protection of the cells against the deleterious effects triggered by toxin-producing *G. vaginalis* and uropathogenic *E. coli*. Furthermore, these compounds crossed the cell membrane to kill the pre-internalized microbial pathogens. In addition, all *L. gasseri* and *L. crispatus* cells exhibited another non-strain specific activity which inhibited the association of microbial pathogens with cervix epithelial cells with varying efficiency, partially protecting the cells against lysis and detachment triggered by toxin-producing *G. vaginalis* and uropathogenic *E. coli*. Our results provide evidence of strain-level specificity for certain antimicrobial properties among cervicovaginal *L. gasseri* and *L. crispatus* strains, indicating that the presence of a particular species in the vaginal microbiota is not sufficient to determine its benefit to the host. A full repertory of antimicrobial properties should be evaluated in choosing vaginal microbiota-associated *Lactobacillus* isolates for the development of live biotherapeutic strategies.

## Introduction

Vaginal dysbiosis (De Seta et al., 2019), bacterial vaginosis (BV) (Onderdonk et al., 2016) and urinary-tract infections (UTIs) (Foxman, 2014) are major health problems that are difficult to treat and highly recurrent. BV is the most common cervicovaginal condition among women of childbearing age and is associated with adverse reproductive health outcomes, including preterm birth and low birth weight, as well as an increased risk of acquiring or transmitting sexually transmitted infections, including HIV-1 (Onderdonk et al., 2016). Clinically, UTIs are differentiated into lower (cystitis) and upper UTIs (pyelonephritis). Nearly half of all women will experience a UTI in their lifetime and 20–30% of those with acute cystitis will have a recurrence within 3 to 4 months (Foxman, 2014). A recent study showed that bladder exposure to BV-associated *Gardnerella vaginalis* can activate uropathogenic *Escherichia coli* (UPEC) from latent bladder epithelial reservoirs, thus triggering the recurrence of cystitis (Gilbert et al., 2017).

The human urogenital tract is colonized by a large diversity of microorganisms, representing a complex microbial ecosystem in which host and microbes exist in homeostasis (Whiteside et al., 2015; Smith and Ravel, 2017). The resilience of this ecosystem greatly depends on environmental exposure and behavioral factors, as well as a range of host factors, which vary between individuals, during life and geographically. Genomic and functional comparison of vagina- or bladder-associated bacterial strains are suggesting that these two body sites are microbiologically linked (Thomas-White et al., 2018). Cervicovaginal microbiota can affect women’s health since microbiota dominated by *Lactobacillus* spp. (Ravel et al., 2011; Romero et al., 2014) has been shown to be associated with a reduced risk of microbial infections (Amabebe and Anumba, 2018). *Lactobacillus crispatus*, *Lactobacillus gasseri*, *Lactobacillus jensenii*, and *Lactobacillus iners* are commonly found in the cervicovaginal microbiota of apparently healthy women (Antonio et al., 1999). Molecular analyses have identified at least five major types of cervicovaginal microbiota, called community state types (CSTs), which differ in bacterial composition and relative abundance. Four are dominated by either *L. crispatus* (CST I), *L. gasseri* (CST II), *L. iners* (CST III), or *L. jensenii* (CST V) (Ravel et al., 2011; Romero et al., 2014). The fifth CST (CST IV) comprised facultative anaerobic bacteria, including *G. vaginalis, Atopobium vaginae*, and *Megasphaera* spp., among others, and resembles the composition of the vaginal microbiota associated with BV (Ravel et al., 2011; Romero et al., 2014). A meta-analysis of clinical studies has demonstrated that women with low-*Lactobacillus* CST IV cervicovaginal microbiota are at increased risk of *Prevotella bivia*, *G. vaginalis*, *Chlamydia trachomatis* and human papillomavirus infections, whereas women with *Lactobacillus*-dominated cervicovaginal microbiota are at lower risk (Tamarelle et al., 2019). However, the association between the risk of UTIs and the composition of the vaginal microbiota is still unclear (Whiteside et al., 2015).

Probiotic *Lactobacillus*-based therapeutics to treat BV and UTIs are available (Foxman and Buxton, 2013; Geerlings et al., 2014; Reid, 2017). Moreover, “live biotherapeutic” drugs are being developed under the FDA regulatory framework (FDA, 2016). The *in vitro* probiotic antimicrobial properties (FAO/WHO, 2002; Hill et al., 2014) associated with whole bacterial cells, secreted metabolites or released compounds of *L. gasseri* and *L. crispatus*. urogenital isolates have been well documented (Servin, 2004; Spurbeck and Arvidson, 2011; Petrova et al., 2013; Smith and Ravel, 2017). Clinical trials have confirmed the therapeutic interest of some of these strains to treat dysbiosis, BV and UTIs (MacPhee et al., 2010; Hanson et al., 2016; De Vrese et al., 2019; van de Wijgert and Verwijs, 2019). Here, we aimed to evaluate the distribution of a range of *in vitro* antimicrobial activities against BV-associated *P. bivia* and *G. vaginalis*, UPEC, and recurrent cystitis and infection-related preterm labor-associated *E. coli* in cervicovaginal strains of *L. gasseri* and *L. crispatus*. Vaginal (Onderdonk et al., 2016) and urinary tract (Hannan et al., 2012; Servin, 2014; Flores-Mireles et al., 2015; Mobley, 2016; Tamadonfar et al., 2019) bacterial pathogens have evolved sophisticated virulence mechanisms, including, among others, flagella, adhesins, toxins, and siderophores associated with biofilm formation, epithelial cell colonization and invasion, and cytotoxic activities. Thus, our analysis of the repertory of probiotic antimicrobial activities of urogenital strains *L. gasseri and L. crispatus* included bactericidal and bacteriostatic effects on free, adhering, or internalized pathogens and biofilm disrupting activity; inhibition of pathogen association with cervix epithelial cells by competition, exclusion, or displacement; and protective properties against the deleterious cellular effects of specific pathogen toxins.

## Materials and Methods

### *Lactobacillus* Strains

The collection of twenty two *Lactobacillus* strains isolated from cervicovaginal samples of healthy women were from strains collection of UMR-S 510 Inserm (Faculty of Pharmacy, University of Paris-Sud, Châtenay-Malabry. 92296. France) (Atassi et al., 2006a, b). Bacteria are identified by biochemical test (Kandler and Weiss, 1986) and analysis of *tuf* sequences (Chavagnat et al., 2002). The human cervicovaginal *L. gasseri* strain ATCC 9857 was obtained from American Type Culture Collection (Manassas, VA, United States). The human cervicovaginal *L. gasseri* strain KS 120.1 was provided by ProbioSwiss SA (Zurich, Switzerland). The human cervicovaginal *L. crispatus* strain CTV-05 was kindly provided by Pr. P. B. Heczko (Department of Microbiology, Jagiellonian University Medical College, Krakow, Poland).

All *Lactobacillus* strains were grown in De Man, Rogosa, Sharpe (MRS) broth (Gibco, Thermo Fisher Scientific, Paris, France) for 18 h at 37°C with 5% CO_2_ (Atassi et al., 2006a). For assays, 18-h cultures adjusted to 10^9^ CFU/ml were used. Bacterial cells and cell-free culture supernatants (CFCSs) were obtained by centrifuging the *Lactobacillus* cultures at 10,000 × *g* for 30 min at 4°C. Separated bacterial cells were washed three times with sterile MRS and resuspended in fresh MRS. CFCSs were passed through a sterile 0.22-mm Millex GS filter unit (Millipore, Molsheim, France). The absence of cells from CFCSs was verified using a colony-count assay.

### Bacterial Pathogens

Human *G. vaginalis* strain 594 of Gardner and Dukes (DSM 4944, ATCC 14018) was obtained from Deutsche Sammlung von Mikroorganismen und Zellkulturen (Braunschweig, Germany). Human clinical isolate *P. bivia* strain CI-1 was provided by the Department of Obstetrics and Gynecology, Zurich University Hospital (Switzerland). Strains were grown on *Gardnerella* agar plates purchased from BioMerieux (Marcy-l’Etoile, France, France). The agar plates were incubated under anaerobic conditions, using a sealed anaerobic jar (Becton Dickinson, United States), at 37°C for a maximum of 36 h. Before use, *G. vaginalis* and *P. bivia* strains were sub-cultured in Brain-Heart-Infusion (BHI) medium (Gibco, Thermo Fisher Scientific) supplemented with yeast extract, maltose, and horse serum, under anaerobic conditions, using a sealed anaerobic jar, at 37°C (Atassi et al., 2006a).

The human prototypic wildtype pyelonephritis-associated *E. coli* strain CFT073 (UPEC CFT073) (Mobley et al., 1990) was provided by Pr. Harry Mobley (Department of Microbiology and Immunology, University of Michigan Medical School, Ann Arbor, MI, United States). The human prototypic wildtype *E. coli* strain IH11128 (Nowicki et al., 1987), a member of the diffusely adhering *E. coli* family that expresses Afa/Dr. adhesins and associated with recurrent cystitis and infection-related preterm labor, was provided by Dr. Bogdan J. Nowicki (Department of Obstetrics and Gynecology, Meharry Medical College, Nashville, TN, United States). The strains were maintained on Luria-Bertani (LB) plates. Before infection, bacteria were grown in LB broth Miller (Gibco, Thermo Fisher Scientific) at 37°C (Atassi et al., 2006b).

### Killing Assay

A colony count assay was performed to measure the effect on viability of the test bacterial pathogens (10^8^ CFU/ml) incubated in the appropriate culture medium described above, with or without an 18-h *Lactobacillus* culture (adjusted to 10^9^ CFU/ml) or CFCS at 37°C. Incubations were conducted in BHI or Dulbecco’s modified Eagle’s minimum essential medium (DMEM) (Gibco), as indicated. Aliquots were removed initially and at predetermined intervals, serially diluted, and plated on appropriate bacterial media, described above, to determine the bacterial colony counts of the pathogens. According to the guidelines of the Clinical and Laboratory Standards Institute (Standards, 1999), the minimum bactericidal effect is conventionally defined as a 3 log_10_ CFU/ml (MBE_99_._9__%_) decrease in the number of viable bacteria. Here, we used a more restrictive criterion, using an MBE_99_._99__%_ value, defined as a reduction of the viable bacterial count of 4 log_10_ CFU/ml.

### Treatment With Catalase

The *Lactobacillus* CFCSs were treated prior to the assay at 37°C for 1 h, with or without catalase (5 μg/ml) (Sigma-Aldrich, L’Isle d’Abeau Chesnes, France), to determine the part of the killing effect dependent on hydrogen peroxide.

### Growth Inhibition Assay

The effect on growth of the bacterial pathogens was measured by incubating a test bacterial pathogen (10^6^ CFU/ml) in its appropriate culture medium with an aliquot of CFCS of an 18-h *Lactobacillus* culture (adjusted to 10^9^ CFU/ml). Under all experimental conditions, bacterial growth was quantified by optical density at 620 nm on a Tecan GENios Microplate Reader (Tecan, Trappes, France).

### Biofilm Assay

Uropathogenic *Escherichia coli* CFT073 forming biofilm (Luterbach et al., 2018) was used as a pathogen test strain. Bacteria were grown (10^8^ CFU/ml) in LB in wells of a 96-well plate for 72 h at 37°C. The biofilms were gently washed twice with sterile phosphate-buffered saline (PBS) to remove non-adherent cells. For determination of activity of *Lactobacillus* CFCSs on pre-formed biofilms, CFCS (250 μl) of 18 h culture adjusted to 10^9^ CFU/ml) was added in the presence of DMEM. Quantification of the biofilms was determined by Crystal Violet staining (addition of 0.5% Crystal Violet per well, incubation for 5 min, and then discarded) and measuring optical density at 600 nm. The remaining numbers of viable biofilm-associated bacteria after treatment were determined by scraping the biofilms, dispersing the cells in PBS, and plating dilutions for bacterial colony counts.

### Cell Culture

Human cervical epithelial HeLa cells were seeded (5 × 10^5^ cells per well) in culture plates (TPP, Dominique Dutscher SAS, Brumath, France) and were cultured at 37°C in 5% CO2/95% air in RPMI 1640 with L-glutamine (21875-034 – Gibco, Thermo Fisher Scientific), supplemented with 10% heat-inactivated (30 min, 56°C) fetal calf serum (FCS; Gibco, Thermo Fisher Scientific) (Atassi et al., 2006a, b; Lievin-Le Moal et al., 2011). Cells were used for infection assays at post-confluence (7 days in culture).

### Killing Assay in Infected Cells

Bacterial pathogens associated with HeLa cells were quantified by infecting the confluent cell monolayers for 60 min with a test bacterial pathogen (10^8^ CFU/ml). The plates were then washed five times with sterile PBS to remove non-adhering bacteria and then incubated for 3 h with *Lactobacillus* CFCSs in DMEM.

Bacterial internalized into untreated and *Lactobacillus*-treated cells were quantified using the gentamicin assay. Confluent cell monolayers were infected for 120 min with a test bacterial pathogen (10^8^ CFU/ml). Infected cells were washed with sterile PBS and incubated for 60 min with cell culture medium containing 100 g/ml gentamicin (Invitrogen, Thermo Fisher Scientific), an antibiotic that does not cross the cell membrane and which rapidly kills the cell membrane-associated bacteria but not those located inside the cells. After washing four times with sterile PBS, the cell monolayers were then incubated with a CFCS for 3 h in the presence of DMEM.

All cell infections were conducted at 37°C in 10% CO_2_/90% air. At the end of each assay, the cells were washed three times with sterile PBS and lysed with sterile H_2_O. Dilutions were plated on the appropriate culture medium to determine the number of viable cell-associated or internalized bacteria by colony counts.

### Competition, Exclusion, and Displacement Assays

For the competition assay, confluent HeLa cell monolayers were incubated for 3 h with a test bacteria pathogen (10^8^ CFU/ml), with or without *Lactobacillu*s cells (10^8^ CFU/ml), in DMEM. For the exclusion assay, confluent cell monolayers were incubated for 3 h with or without *Lactobacillu*s cells (10^8^ CFU/ml) in DMEM, washed three times with sterile PBS, and then infected for 3 h with a test bacterial pathogen (10^8^ CFU/ml). For the displacement assay, confluent cell monolayers were incubated for 3 h with a test bacterial pathogen, washed three times with sterile PBS, and then sub-cultured with or without *Lactobacillu*s cells (10^8^ CFU/ml) for 3 h in DMEM. All incubations were conducted at 37°C in 10% CO_2_/90% air. The numbers of viable cell-associated pathogenic bacteria were determined by lysing the cells in sterile H_2_O and plating dilutions on the appropriate medium for bacterial colony counting. Each assay was conducted in triplicate, with three successive cell passages. Results are expressed as the percent of adhering bacteria.

### Cytoprotection Assays

The cytoprotective effect of pre-adhering *Lactobacillus* cells was assessed by pre-colonization of the confluent HeLa cell monolayers by *Lactobacillus* cells (10^8^ CFU/ml, 3 h of incubation), washing away the non-adherent bacteria, and infection with a test bacterial pathogen (10^8^ CFU/ml). The cytoprotective effect of CFCSs of 18-h *Lactobacillus* cultures (adjusted to 10^9^ CFU/ml) was tested by incubating confluent cell monolayers with a test bacterial pathogen (10^8^ CFU/ml), with or without CFCSs in DMEM. The attached HeLa cells were counted by phase-contrast light microscopy. Cells were examined using an Aristoplan microscope (Leitz, Germany) with epifluorescence (Plan Aprochromat 100X/1.32–0.6 oil-immersion objective). For each sample more than 15 random microscopic fields were examined. The source of the images was hidden from the people counting the number of attached cells in order to eliminate any possible bias. Photographic images were resized, organized and labeled using Adobe Photoshop software (San Jose, CA, United States).

### Analysis

Results are expressed as the means ±standard error of the mean. Student’s *t*-test was performed for statistical comparisons.

## Results

### Killing Activity by Direct Contact

**FIGURE 1:**
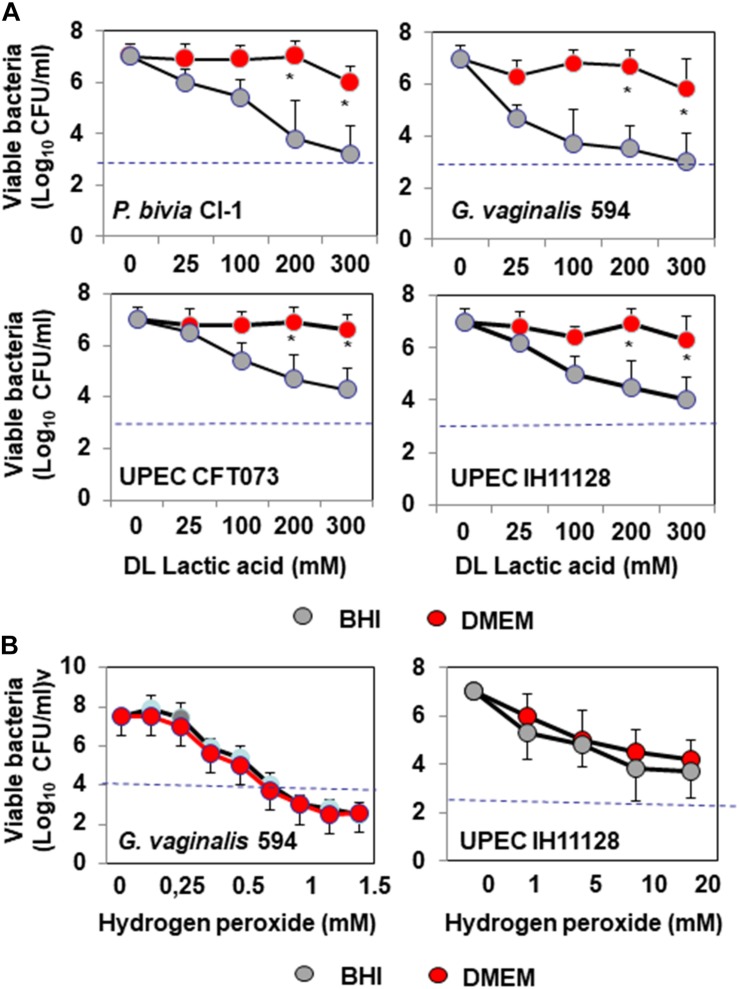
*In vitro* lactic acid-dependent and -independent killing activity by direct contact against BV-associated *P. bivia* CI-1 and *Gardnerella. vaginalis* 594 strains, pyelonephritis-associated *E. coli* strain CFT073, and recurrent cystitis- and preterm labor-associated *E. coli* strain IH11128. **(A)** Concentration-dependent killing activity of DL-lactic acid in BHI or DMEM. **(B)** Concentration-dependent killing activity of hydrogen peroxide in the presence of BHI or DMEM. Each value shown is the mean ±SD from three experiments. Student *t*-test, ^∗^*p* < 0.01 compared to BHI. The dotted line shows the MBE_99_._99__%_ value, defined as a reduction in the viable cell count of 4 log_10_.

We assessed the antibiotic-like killing activities of isolates dependent or not of secreted lactic acid using a previously described method that allows discrimination of the lactic acid-dependent and -independent activities of *Lactobacillus* against *Salmonella enterica* typhimurium (Fayol-Messaoudi et al., 2005). DL-lactic acid at pH 4.5 in the presence of BHI decreased the viability of BV-associated *P. bivia* CI-1 and *G. vaginalis* 594, pyelonephritis-associated CFT073 (UPEC CFT073), and recurrent cystitis and preterm labor-associated IH11128 (UPEC IH11128) in a concentration-dependent manner (Figure 1A). Adding DMEM to the medium abolished the antimicrobial activity of DL lactic acid against urogenital microbial pathogens without modifying the pH (pH 4.5 ± 0.4). In addition, the concentration-dependent killing activity of hydrogen peroxide was not affected by adding DMEM (Figure 1B).

**FIGURE 2:**
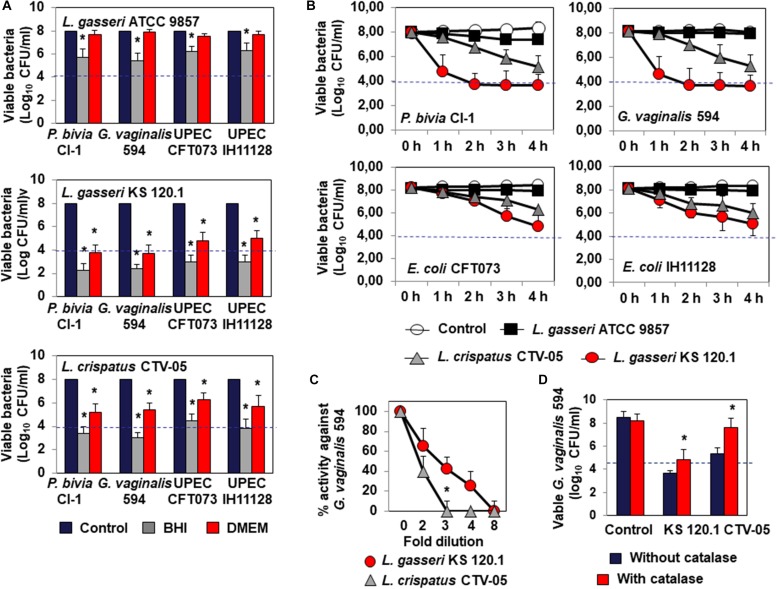
Characteristics of the killing activity by direct contact of *L. gasseri* ATCC 9857, *L. gasseri* KS 120.1, and *L. crispatus* CTV-05 strains against BV-associated *P. bivia* CI-1 and *G. vaginalis* 594 strains, pyelonephritis-associated *E. coli* strain CFT073, and recurrent cystitis- and preterm labor- associated *E. coli* strain IH11128. **(A)** Lactic acid-dependent (BHI) and -independent (DMEM) killing activities exerted by each *Lactobacillus* strain in co-culture conditions. **(B)** Time-course of lactic acid-independent killing activity of *L. gasseri* ATCC 9857, *L. gasseri* KS 120.1, and *L. crispatus* CTV-05 CFCSs. **(C)** Concentration-dependent killing activity of *L. gasseri* KS 120.1 and *L. crispatus* CTV-05 CFCSs. **(D)** Effect of catalase treatment on the killing activity of *L. crispatus* CTV-05 CFCS. In **(A)**, killing activity was determined after 4 h of direct contact with *Lactobacillus* cultures (18 h of culture adjusted to 10^8^ CFU/ml *Lactobacillus* bacteria). In **(A)**, the dotted line shows the MBE_99_._99__%_ value, defined as a reduction in the viable cell count of 4 log_10_ CFU/ml. In **(C,D)**, killing activity was determined in the presence of DMEM after 4 h of direct contact. Each value shown is the mean ±SD from three experiments. In **(A)**, Student *t*-test, ^∗^*p* < 0.01 compared to control. In **(B)**, Student *t*-test, ^∗^*p* < 0.01 at 3 and 4 h. In **(C,D)**, Student *t*-test, ^∗^*p* < 0.01 compared to control.

*Lactobacillus gasseri* ATCC 9857 CFCS exerted killing activity against *P. bivia* CI-1 and *G. vaginalis* 594 (2.43 ± 0.72 log_10_ and 2.65 ± 0.83 log_10_ CFU/ml decrease in viability, respectively) and UPEC CFT073 and IH11128 (1.76 ± 0.56 and 1.84 ± 0.44 log_10_ CFU/ml decrease in viability, respectively), which was dependent on lactic acid, as it was completely abolished by the addition of DMEM (Figure 2A). *L. gasseri* KS 120.1 CFCS also exerted killing activity against *P. bivia* CI-1 and *G. vaginalis* 594 (6.43 ± 0.61 log_10_ and 6.35 ± 0.58 log_10_ CFU/ml decrease in viability, respectively), and UPEC CFT073 and IH11128 (5.33 ± 0.56 and 5.04 ± 0.48 log_10_ CFU/ml decrease in viability, respectively), which largely persisted in the presence of DMEM (*P. bivia* CI-1: 4.20 ± 0.6, *G. vaginalis* 594: 4.30 ± 0.41, UPEC CFT073: 3.21 ± 0.67, and UPEC IH11128: 3.00 ± 0.47 log_10_ CFU/ml decrease in viability) (Figure 2A). Similarly, *L. crispatus* CTV-05 CFCS also exerted killing activity, but to a lesser extent (*P. bivia* CI-1: 4.61 ± 0.53, *G. vaginalis* 594: 5.05 ± 0.42, UPEC CFT073: 3.51 ± 0.49, and UPEC IH11128: 4.23 ± 0.67 log_10_ CFU/ml decrease in viability), which was diminished by approximately one half in the presence of DMEM (*P. bivia* CI-1: 2.80 ± 0.68, *G. vaginalis* 594: 2.60 ± 0.41, UPEC CFT073: 1.72 ± 0.67, and UPEC IH11128: 2.31 ± 0.47 log_10_ CFU/ml decrease in viability). We next measured lactic acid-independent killing activity in the presence of DMEM over 4 h. *L. gasseri* KS 120.1 CFCS achieved maximum efficacy against *P. bivia* CI-1 and *G. vaginalis* 594 after 2 h, whereas the activity against UPEC CFT073 and UPEC IH11128 developed more slowly (Figure 2B). In contrast, the lactic acid-independent killing activity of *L. crispatus* CTV-05 CFCS against the four bacterial pathogens developed slowly (Figure 2B).

The lactic acid-independent killing activities of *L. gasseri* KS 120.1 and *L. crispatus* CTV-05 CFCSs displayed the concentration-dependent pharmacodynamics characteristic of antimicrobial agents (Levison and Levison, 2009). The lactic acid-independent killing activities of *L. gasseri* KS 120.1 and *L. crispatus* CTV-05 CFCSs were concentration-dependent, with the CFSC of *L. gasseri* KS 120.1 maintaining potency at higher dilutions than that of *L. crispatus* CTV-05 (Figure 2C). In addition, the lactic acid-dependent and -independent killing activity of *L. crispatus* CTV-05 CFCS was abolished after catalase treatment (Figure 2D). The lactic acid-independent killing activity of *L. gasseri* KS 120.1 CFCS was only diminished by 1.2 ± 0.4 log_10_ after catalase treatment (Figure 2D), in agreement with a previous report (Atassi et al., 2006b).

### Bacteriostatic Activity

**FIGURE 3:**
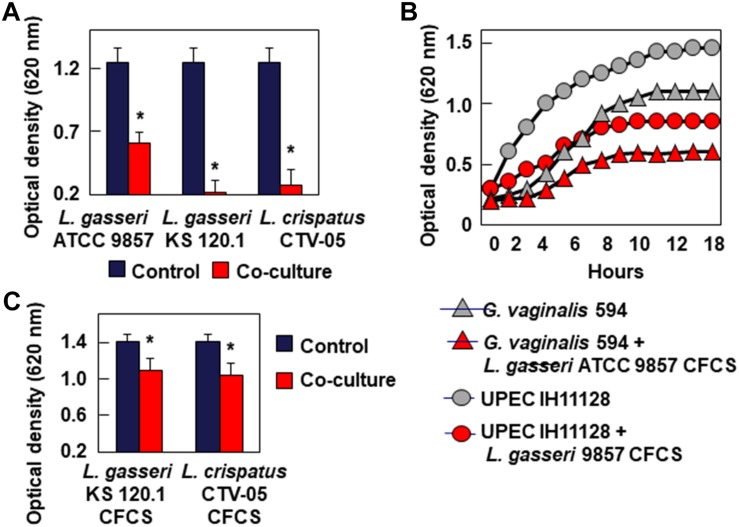
Inhibition of growth of BV-associated *G. vaginalis* 594, and recurrent cystitis- and preterm labor-associated *E. coli* strain IH11128. **(A)** Inhibition of *G. vaginalis* 594 growth after 24 h of co-culture with *L. gasseri* ATCC 9857, *L. gasseri* KS 120.1, or *L. crispatus* CTV-05 strains. **(B)** Inhibition of *G. vaginalis* 594 and UPEC IH11128 growth during a time-course of co-culture with *L. gasseri* ATCC 9857 CFCS. **(C)** Inhibition of UPEC IH11128 growth after 24 h of co-culture in the presence of 8-fold diluted *L. gasseri* KS 120.1, and *L. crispatus* CTV-05 CFCSs. Each value shown is the mean ±SD from three experiments. Student *t*-test, ^∗^*p* < 0.01 compared to control.

Testing bacteriostatic activity against *G. vaginalis* 594 and UPEC HI11128 as the pathogen test strains showed the following results After 24 h of contact, *L. gasseri* ATCC 9857 CFCS inhibited the growth of *G. vaginalis* DSM 594 and UPEC HI11128, whereas *L. gasseri* KS 120.1 and *L. crispatus* CTV-05 CFCSs completely abolished the growth of the two pathogens (Figure 3A). The bacteriostatic activity of *L. gasseri* ATCC 9857 CFCS against *G. vaginalis* DSM 594 and UPEC HI11128 was time-dependent (Figure 3B). The abolition of growth by *L. gasseri* KS 120.1 and *L. crispatus* CTV-05 CFCSs after 24 h of contact (Figure 3A) could potentially be the result of their killing activity (Figure 2). However, we observed significant inhibition of UPEC IH11128 growth (Figure 3C) using an eight-fold dilution of *L. gasseri* KS 120.1 and *L. crispatus* CTV-05 CFCSs, which no longer had killing activity (Figure 2C).

### Activity Against Biofilms

Bacterial pathogens associated with urogenital infections often form biofilms at the epithelial cell surface (Hannan et al., 2012; Tamadonfar et al., 2019; Verstraelen and Swidsinski, 2019). Crystal Violet uptake, as measured by the OD_600_, showed that the size of pre-formed UPEC CFT073 biofilms (Untreated: 0.65 ± 0.05 OD_600 nm_) was not modified after 48 h of treatment with *L. gasseri* ATCC 9857, *L. gasseri* KS 120.1, or *L. crispatus* CTV-05 CFCSs (0.62 ± 0.05, 0.61 ± 0.12, and 0.71 ± 0.09 OD_600 nm_, respectively). Moreover, the number of viable UPEC CFT073 bacteria present within the pre-formed biofilms (Untreated: 8.59 ± 0.25 log_10_ CFU/ml) were not significantly decreased, only by approximately 1 log_10_ CFU/ml by *L. gasseri* ATCC 9857, *L. gasseri* KS 120.1, and *L. crispatus* CTV-05 CFCSs (7.69 ± 0.45, 7.48 ± 0.92, and 7.68 ± 0.98 log_10_ CFU/ml, respectively).

### Competition, Exclusion, and Displacement Activities Against Epithelial Cell Colonization

**FIGURE 4:**
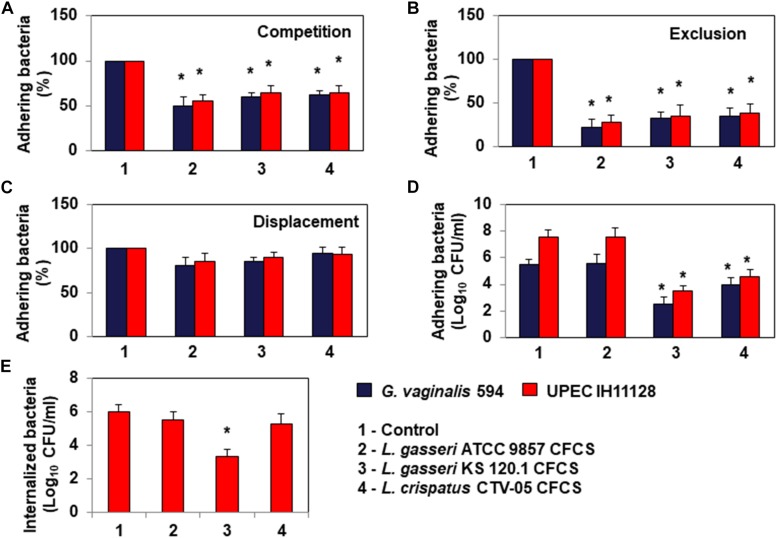
Antagonistic activities of *Lactobacillus* cells and CFCSs against BV-associated *G. vaginalis* 594 and recurrent cystitis- and preterm labor-associated *E. coli* IH11128 adhering to HeLa cells. **(A)** Inhibition under competition conditions. **(B)** Inhibition under exclusion conditions. **(C)** Lack of inhibition under displacement conditions. **(D)** Killing of pre-adhering UPEC IH11128. **(E)** Killing of pre-internalized UPEC IH11128. In **(A–C)**, 100% adhesion corresponds to 5.1 ± 0.5 CFU/ml for *G. vaginalis* 594 and 7.6 ± 0.4 CFU/ml for *E. coli* IH11128. Competition, exclusion and displacement experimental condition are described in section “Materials and Methods”. Each value shown is the mean ±SD from three experiments. Student *t*-test, ^∗^*p* < 0.01 compared to control.

Bacterial pathogens associated with BV or UTIs can often colonize cervicovaginal and urinary tract epithelia (Hannan et al., 2012; Servin, 2014; Flores-Mireles et al., 2015; Mobley, 2016; Onderdonk et al., 2016). We thus evaluated whether *L. gasseri* ATCC 9857, *L. gasseri* KS 120.1 and *L. crispatus* CTV-05 cells can inhibit HeLa cell colonization by *G. vaginalis* 594 and UPEC IH11128 (Figure 4). Under conditions of competition, in which the pathogens and *Lactobacillus* cells were co-incubated with the HeLa cells, there was a significant decrease of adhesion of *G. vaginalis* 594 and UPEC IH11128 in the presence of *L. gasseri* ATCC 9857 (50.2 ± 7.2 and 45.5 ± 5.4% decrease, respectively), *L. gasseri* KS 120.1 (40.5 ± 4.2 and 35.8 ± 8.2% decrease, respectively), or *L. crispatus* CTV-05 cells (38.6 ± 5.5 and 35.1 ± 7.4% decrease, respectively) (Figure 4A). Under conditions of exclusion, the adhesion of *G. vaginalis* 594 and UPEC IH11128 to HeLa cells was decreased in the presence of pre-adhering *L. gasseri* ATCC 9857 (78.1 ± 9.3 and 72.8 ± 8.6% decrease, respectively), *L. gasseri* KS 120.1 (68.0 ± 7.7 and 65.8 ± 12.1% decrease, respectively), or *L. crispatus* CTV-05 cells (65.5 ± 9.8 and 62.4 ± 12.1% decrease, respectively) (Figure 4B). Under conditions of displacement, the level of pre-adhering pathogens was not affected by the addition of *L. gasseri* ATCC 9857, *L. gasseri* KS 120.1, or *L. crispatus* CTV-05 cells (Figure 4C).

Evaluating the ability of *L. gasseri* KS 120.1 and *L. crispatus* CTV-05 CFCSs to kill pathogens pre-associated with Hela cells, we found: The level of viable *G. vaginalis* 594 and UPEC IH11128 pre-adhering onto HeLa cells was reduced after treatment of pre-infected cells with *L. gasseri* KS 120.1 CFCS (3.0 ± 0.61 and 4.02 ± 0.22 log_10_ CFU/ml decrease of viable pre-adhering bacteria, respectively) and *L. crispatus* CTV-05 CFCS (1.49 ± 0.45 and 3.05 ± 0.29 log_10_ CFU/ml decrease of viable pre-adhering bacteria, respectively) (Figure 4D). Consistent with the absence of lactic acid-independent killing activity of *L. gasseri* ATCC 9857 CFCS (Figure 2), *L. gasseri* ATCC 9857 CFCS treatment in the presence of DMEM failed to affect the level of viable, pre-adhering *G. vaginalis* 594 or UPEC IH11128 (Figure 4D).

Uropathogenic *Escherichia coli* internalization into cells lining the urothelium is an important step of pathogenesis because it creates a reservoir of dormant bacteria (Hannan et al., 2012; Servin, 2014; Flores-Mireles et al., 2015; Mobley, 2016). Thus, we evaluated the ability of *L. gasseri* KS 120.1 and *L. crispatus* CTV-05 CFCSs to kill UPEC IH11128 pre-internalized within Hela cells. *L. gasseri* KS 120.1 CFCS, in the presence of DMEM, decreased the viability of pre-internalized UPEC IH11128 cells (2.65 ± 0.461 log_10_ CFU/ml decrease of viable pre-internalized bacteria) (Figure 4E). In contrast, given that *L. crispatus* CTV-05 CFCSs exerts lactic acid-independent killing activity against free and adhering UPEC IH11128 (Figures 2, 4D, respectively), it failed to decrease the level of pre-internalized UPEC IH11128 in the presence of DMEM (Figure 4E). Consistent with *L. gasseri* ATCC 9857 CFCS lacking lactic acid-independent killing activity against free and adhering UPEC IH11128 (Figures 2, 4D, respectively), it also did not decrease the level of pre-internalized UPEC IH11128 in the presence of DMEM.

### Cytoprotective Effect Against Bacterial Toxins Produced Cell-Detachment

**FIGURE 5:**
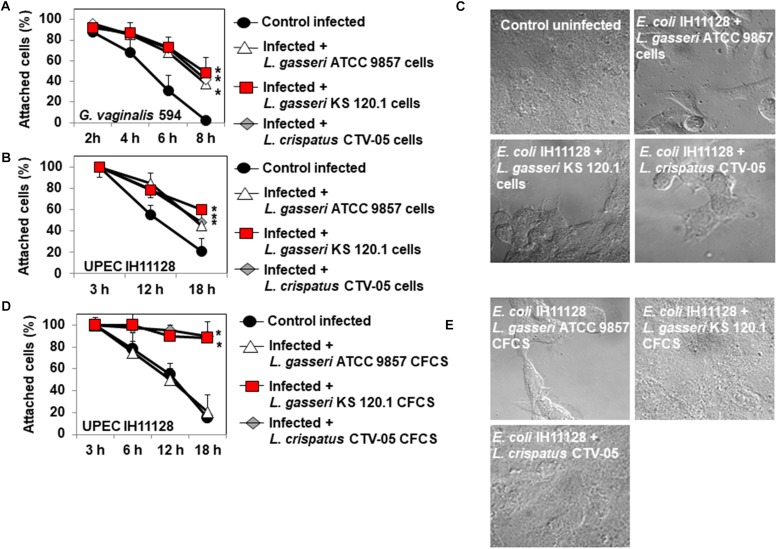
Protective effect of cells and CFCSs of *L. gasseri* ATCC 9857, *L. gasseri* KS 120.1, or *L. crispatus* CTV-05 strains against the cytotoxic activities of BV-associated *G. vaginalis* 594 and preterm labor and recurrent cystitis-associated *E. coli* IH11128. **(A)** Quantification of time-dependent *G. vaginalis* 594-induced cell-detachment in the presence, or not, of pre-colonizing *Lactobacillus* cells. **(B)** Quantification of time-dependent UPEC IH11128-induced cell detachment, in the presence, or not, of pre-colonizing *Lactobacillus* cells. **(C)** Micrographs illustrating the partial inhibition of UPEC IH11128-induced cell detachment in the presence of pre-colonizing *Lactobacillus* cells. **(D)** Quantification of cell detachment in cell monolayers infected with UPEC IH11128, showing the total inhibition of cell detachment in the presence *L. gasseri* KS 120.1 or *L. crispatus* CTV-05 CFCSs and the lack of inhibition in the presence of *L. gasseri* ATCC 9857 CFCS. **(E)** Micrographs illustrating the entire inhibition of UPEC IH11128-induced cell detachment in the presence *L. gasseri* KS 120.1 or *L. crispatus* CTV-05 CFCSs. Phase-contrast micrographs are representative of two separate experiments. The number of attached cells was monitored by phase-contrast light microscopy. Each value shown is the mean ±SD from three experiments. Student *t*-test, ^∗^*p* < 0.01 compared to *G. vaginalis* 594 or UPEC IH11128.

Epithelial exfoliation is a hallmark of both BV (Amegashie et al., 2017) and UTIs (Mulvey et al., 2000). *G. vaginalis* 594, producing the cytotoxin, vaginolysin (Yeoman et al., 2010), and UPEC IH11128, producing the Sat toxin (Lievin-Le Moal et al., 2011), are often used as pathogen test strains. *G. vaginalis* 594-infected HeLa cell monolayers on glass slides showed time-dependent disappearance of the cells, with only a small number of cells still attached 8 h post-infection (PI) (2.4 ± 1.7% of the cells remained attached) (Figure 5A). When the confluent HeLa cell monolayers were pre-colonized by *Lactobacillus* cells, there was a partial but significant decrease in *G. vaginalis* 594-induced cell-detachment (*L. gasseri* ATCC 9857 cells: 38.1 ± 15.2%, *L. gasseri* KS 120.1 cells: 48.4 ± 7.9%, and *L. crispatus* CTV-05 CFCS: 42.5 ± 6.1% of the cells were still attached 8 h post-infection) (Figure 5A).

For UPEC IH11128, 20.6 ± 5.2% of the HeLa cells were still attached to the glass slide after 18 h of infection (Figure 5B). There was partial protection against UPEC IH11128-induced cell-detachment when HeLa cells were pre-colonized with *Lactobacillus* cells (*L. gasseri* ATCC 9857 cells: 45.1 ± 9.2%, *L. gasseri* KS 120.1 cells: 60.4 ± 7.9%, and *L. crispatus* CTV-05 CFCS: 48.5 ± 6.1% of the cells were still attached at 18 h post-infection) (Figures 5B,C). We then examined whether the above observed killing of cell-associated UPEC IH11128 by *L. gasseri* KS 120.1 and *L. crispatus* CTV-05 CFCSs in the presence of DMEM improves cell protection. When confluent HeLa cell monolayers were infected by UPEC IH11128 in the presence of *L. gasseri* KS 120.1 or *L. crispatus* CTV-05 CFCSs and DMEM, only a small number of cells had detached by 18 h PI (98 ± 3.1% and 88 ± 6.8% of cells remained attached, respectively) (Figure 5D). As expected, the *L. gasseri* ATCC 9857 CFCS, which lacks lactic acid-independent killing activity (Figure 2), showed no protective effect against UPEC IH11128-induced cell-detachment in the presence of DMEM (20.2 ± 6.6% of cells remained attached) (Figures 5D,E).

### Expression of Killing Activity and Inhibition of Pathogen Adhesion in Human Cervocovaginal Microbiota-Associated *L. gasseri* and *L. crispatus* Isolates

**FIGURE 6:**
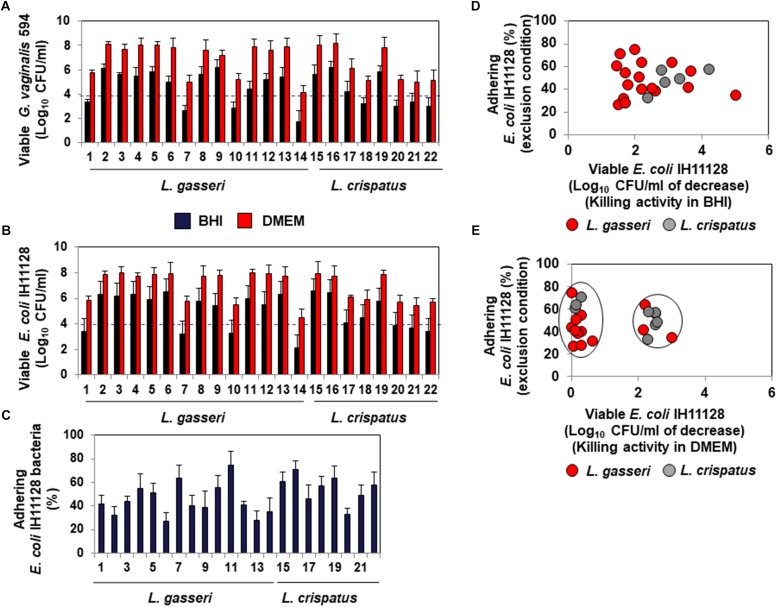
Distribution of the killing activities and adhesion inhibition activity among *L. gasseri* and *L. crispatus* strains isolated from cervicovaginal samples of healthy women. **(A)** Killing activity against BV-associated *G. vaginalis* 594 was determined in the presence of BHI or DMEM. **(B)** Killing activity against recurrent cystitis- and preterm labor- associated UPEC IH11128 was determined in the presence of BHI or DMEM. **(C)** Competitive inhibition activity exerted by *L. gasseri* and *L. crispatus* strains against adhesion of UPEC IH11128 onto cervicovaginal epithelial HeLa cells. **(D)** Strain-by-strain expression of total killing activity (in the presence of BHI) versus inhibition of pathogen adhesion by *Lactobacillus* cells. **(E)** Strain-by-strain expression of killing activity independent of secreted lactic acid (in the presence of DMEM) versus inhibition of pathogen adhesion by *Lactobacillus* cells. In **(A,B)**, the killing activity was measured after 4 h of direct contact with the pathogens (Control: 5 × 10^8^ CFU/ml). The dotted line shows the MBE_99_._99__%_ value, defined as a reduction in the viable cell count of 4 log_10_ CFU/ml. In C, the inhibition of adhesion was measured after 3 h of incubation. 100% UPEC IH11128 adhesion corresponds to 7.1 ± 0.7 CFU/ml. Each value shown is the mean ±SD from three experiments. Graphs in **(D,E)** were constructed by plotting the killing activities (measured in the presence of BHI or DMEM) shown in Figure 1B versus the exclusion inhibition activities shown in Figure 1C.

The lactic acid-dependent and -independent killing activity of a set of fourteen *L. gasseri* and eight *L. crispatus* isolates is depicted in Figures 6A,B, respectively, highlighting the large variation in the potency of the isolates against *G. vaginalis* 594 and UPEC IH11128. Evaluation of the ability of the isolates to inhibit the adhesion of UPEC IH11128 onto human cervicovaginal epithelial HeLa cells by competition showed that cells of almost all isolates were able to prevent adhesion by UPEC IH11128 but with variable efficacy (Figure 6C). Plotting the killing activity in the presence of BHI versus the inhibition of adhesion by competition shows the lactic acid-dependent killing activity and competitive inhibition by *Lactobacillus* cells against UPEC IH11128 adhesion to be two properties that are widely distributed among *L. gasseri* and *L. crispatus* vaginal isolates (Figure 6D). Furthermore, plotting the killing activity measured in the presence of DMEM versus competitive inhibition by *Lactobacillus* cells against UPEC IH11128 adhesion shows that only a small number of *L. gasseri* vaginal isolates expressed lactic acid-independent killing activity dependent on released compound(s) (4 of the 14 isolates: isolates 1, 7, 10 and 14) (Figure 6E). In contrast, the lactic acid-independent killing activity dependent on released compound(s) appeared to be more widely expressed in *L. crispatus* vaginal isolates (5 of the 8 isolates: isolates 17, 18, 20, 21, and 22) (Figure 6E).

## Discussion

This study contributes to our understanding of the protective mechanisms provided by key members of the cervicovaginal microbiota against major microbial pathogens involved in BV and UTIs. We demonstrate that all cervicovaginal microbiota-associated *L. gasseri* and *L. crispatus* strains examined possess non-strain-specific physical and chemical defensive properties and that very few strains express strain-specifically properties. In the urogenital tract, *Lactobacillus* species largely contribute to the defense against microbial pathogens by secreting antimicrobial metabolites, mainly lactic acid, hydrogen peroxide, or proteinaceous or non-proteinaceous compounds (Servin, 2004; Spurbeck and Arvidson, 2011; Smith and Ravel, 2017). *In vivo*, vaginal microbiota-associated *Lactobacillus* species are the main source of the organic acid metabolite, lactic acid (Boskey et al., 2001), which is responsible for acidifying the cervicovaginal tract to a pH of ∼3.8–4.2 (Boskey et al., 1999; O’Hanlon et al., 2013). Moreover, lactic acid in its protonated form exerts broad antimicrobial activity against urogenital microbial pathogens *in vitro* and *in vivo* (Atassi and Servin, 2010; O’Hanlon et al., 2011, 2013; Breshears et al., 2015). Lactic acid has also been shown to permeabilize the bacterial outer membrane (Alakomi et al., 2000) and improve the bactericidal activity of hydrogen peroxide against Gram-negative pathogens *in vitro* (Atassi and Servin, 2010). Here, we show that all strains of *L. gasseri* and *L. crispatus* tested had similar lactic acid-dependent killing activity against the four BV- and UTI-associated bacterial pathogens tested, consistent with the constitutive metabolic nature of lactic acid (Tachedjian et al., 2017). Hydrogen peroxide is secreted mostly by the resident *L. crispatus* strains of the cervicovaginal microbiota of healthy women (Eschenbach et al., 1989; Hillier et al., 1992, 1993). It is an antimicrobial molecule that exerts strong *in vitro* bactericidal activity against urogenital BV-associated *P. bivia*, *G. vaginalis*, and *E. coli* (Klebanoff et al., 1991; Strus et al., 2002, 2006; Atassi and Servin, 2010). Consistent with these findings, we found that 75% of the cervicovaginal *L. crispatus* isolates tested display hydrogen peroxide-dependent killing activity by direct contact, as well as against pre-adhering pathogens. The observed absence of killing of intracellular pathogens by the CFCS of the hydrogen peroxide-producing *L. crispatus* CTV-05 is certainly due to the previously demonstrated very poor transmembrane passage of hydrogen peroxide in cultured cells, including HeLa cells, due to a low membrane permeability close to that of water (Bienert et al., 2006; Lim et al., 2016). Importantly, the effectiveness of the antimicrobial effect of hydrogen peroxide in the vagina has been recently called into question (Tachedjian et al., 2018). First, hydrogen peroxide at the physiological concentrations present in the vagina has no antimicrobial effect against BV-associated microbial pathogens *in vitro* (O’Hanlon et al., 2010). Second, cervicovaginal fluid and semen have the capacity to neutralize its bactericidal activity *in vitro* (O’Hanlon et al., 2010), whereas, they do not affect lactic acid-dependent microbicidal activity (O’Hanlon et al., 2011).

We show that approximately 30% of *L. gasseri* vaginal isolates and *L. gasseri* KS 120.1, but not *L. gasseri* ATCC 9857, have a high level of non-lactic acid-dependent, non-hydrogen peroxide-dependent bactericidal activity against BV-associated bacteria and UPEC due to released compound(s). The observed absence of bactericidal activity of *L. gasseri* ATCC 9857 when tested in the presence of DMEM is in accordance with its previously reported absence of killing activity against UPEC isolates (Charteris et al., 2001) and gonococci (Spurbeck and Arvidson, 2008). We show that the released molecule(s) display the capacity to kill pre-adhering and pre-internalized bacterial pathogens. This is important, as it is well-known that internalized bacterial pathogens in the bladder can constitute an intracellular reservoir of dormant bacteria that can lead to the recurrence of infection following the exfoliation of infected cells from the urothelium (Hannan et al., 2012). Moreover, intracellular UPEC are difficult to eradicate by antibiotics (Blango and Mulvey, 2010) and subinhibitory concentrations of antibiotics enhance the formation of intracellular bacterial communities (Goneau et al., 2015). Thus, our data suggest that only certain strains of microbiota-associated *Lactobacillus* could provide strong protection against UTI recurrence by eliminating internalized UPEC.

A large variety of bacteriocins are encoded in the genomes of lactobacilli in a strain-specific manner (Acedo et al., 2018). *L. gasseri* ATCC 9857 has been shown to inhibit the growth of *E. coli* by compounds resembling gassericin C and D (Charteris et al., 2001). In accordance with this finding, we observed the inhibition of UPEC IH11128 growth by *L. gasseri* ATCC 9857. *L. crispatus* CTV-05 possesses seven class-II and class-III bacteriocin-related proteins (Ojala et al., 2014) and inhibits the growth of pyelonephritis-associated J96 and cystitis-associated R45 *E. coli* strains (Butler et al., 2016). Not surprisingly, we observed that its CFCS decreases the growth of UPEC IH11128. Our observation that KS 120.1 CFCS inhibits the growth of IH11128 confirms and supplements those of previous data study (Atassi et al., 2006b) and indicates the production of bacteriocins or bacteriocin-like molecules by this strain.

Bacterial biofilms are difficult to eradicate by antibiotic treatment (Blango and Mulvey, 2010) and subinhibitory concentrations of antibiotics increase UPEC biofilm formation (Goneau et al., 2015). The observed absence of activity is likely related to the fact that bacterial biofilms are formed by tight bacterial cell-to-cell contacts, making the biofilm matrix an impermeable barrier (Flemming et al., 2016). CFCSs of *L. gasseri* KS 120.1 and *L. crispatus* CTV-05 were unable to kill UPEC CFT073 bacteria within its pre-formed biofilm, despite exerting a killing activity against the free pathogen after direct contact. A limited capacity of *L. crispatus* ATCC 33820 to decrease the area, depth, and density of *G. vaginalis* biofilms has been reported (Saunders et al., 2007). In contrast, *L. crispatus* EX533959VC06 (Machado et al., 2013) and 24-9-7 (Breshears et al., 2015) and *L. gasseri* SF1109 (Zanfardino et al., 2017) are able to inhibit the growth of *G. vaginalis*, *Neisseria gonorrhoeae*, and UPEC within their biofilms. These results show that the capacity to counteract the biofilms of urogenital pathogenic bacteria is variably expressed among urogenital *L. gasseri* and *L. crispatus* strains.

Many strains of *Lactobacillus* spp. have been shown to interfere with bacterial pathogen colonization of urogenital epithelium through the adhesive properties of their bacterial cells (Spurbeck and Arvidson, 2011; Smith and Ravel, 2017). Cells of *L. gasseri* ATCC 9857 (Spurbeck and Arvidson, 2008) and KS 120.1 (Atassi et al., 2006a, b) and *L. crispatus* CTV-05 (Kwok et al., 2006; Antonio et al., 2009; Hemmerling et al., 2010; Butler et al., 2016) display adhesiveness or the capacity to colonize. We found that the cells of the *Lactobacillus* strains examined in this study are able to reduce the colonization of HeLa epithelial cells by *G. vaginalis* and UPEC under competition or exclusion conditions, with varying efficacy. Our observation that *L. gasseri* ATCC 9857 cells can inhibit the adhesion of UPEC IH11128 onto cervicovaginal epithelial cells is in accordance with a report by Spurbeck et al. (Spurbeck and Arvidson, 2008), showing that *L. gasseri* ATCC 9857 cells inhibited the association of *N. gonorrhoeae* with cells of the endometrial epithelial cell line Hec-1-B. *L. crispatus* CTV-05 cells have been shown to decrease the adhesion of pyelonephritis-associated J96 and cystitis-associated R45 *E. coli* onto vaginal epithelial cells (Butler et al., 2016). We similarly found that they are able to inhibit the association of *G. vaginalis* and UPEC with cervicovaginal epithelial cells. Our results showing that *L. gasseri* KS 120.1 cells inhibit the association of *G. vaginalis* and UPEC with epithelial cells under competition and exclusion conditions confirm and complement those of previous studies (Atassi et al., 2006a, b). In contrast, we found that *L. gasseri* ATCC 9857, *L. gasseri* KS 120.1, and *L. crispatus* CTV-05 cells were unable to displace pathogens already adhering to the epithelial cells. This is not surprising, because bacterial pathogens often intimately or irreversibly associate with host target cells using adhesive factors that recognize membrane-associated molecules, acting as specific receptors (Berne et al., 2018).

We observed that pre-adhering *L. gasseri* ATCC 9857 and KS 120.1, and *L. crispatus* CTV-05 cells provide partial protection to the epithelial cells against the cytotoxic effects of the toxins vaginolysin and Sat produced by *G. vaginalis* and UPEC, respectively. Our result with pre-adhering *L. crispatus* CTV-05 cells are in accordance with a recent report showing that they have the capacity to attenuate the cytotoxic activity of pyelonephritis-associated J96 and cystitis-associated R45 *E. coli* expressing α-hemolysin (Butler et al., 2016). The observed partial protection by pre-adhering *Lactobacillus* cells is likely because vaginolysin (Gelber et al., 2008) and Sat (Guignot et al., 2007; Lievin-Le Moal et al., 2011) are secreted by the pathogens and therefore highly accessible to the epithelial cells, such that access cannot be prevented by the pre-adhering bacterial cells. In contrast, our results demonstrate that epithelial cells are fully protected against the deleterious effects of pathogen-secreted toxins if the toxin-producing bacterial pathogens are killed at the luminal domain of epithelial cells by a potent bactericidal activity strain-specifically produced by a *Lactobacillus* strain.

The increasing emergence of multi-drug resistant pathogenic bacteria requires the development of innovative therapeutic strategies (O’Brien et al., 2016; Tamadonfar et al., 2019). Importantly, our results provide evidence of high strain-level specificity of antimicrobial properties among cervicovaginal *L. gasseri* and *L. crispatus* strains, suggesting that the presence of one species in the vaginal microbiota is not sufficient to determine its benefits to the host. The development of biotherapeutic strategies based on *L. gasseri* and *L. crispatus* strains isolated from human vaginal microbiota of healthy women will require that the candidate strains be phenotypically well characterized. In particular, antimicrobial properties should be evaluated to determine whether the candidate strains possess a full repertory of potent antimicrobial properties to counteract the diverse deleterious effects of the targeted microbial pathogens. There is great interest in compounds with strong antibiotic-like activities that are released by *L. gasseri* of the urogenital microbiota for their development and application to women’s health. However, the isolation and identification of such compounds has encountered several technical difficulties due to their small molecular mass and relative instability. New technologies, such as imaging mass spectrometry, hold promise for the future objective of identifying and designing more stable effective molecules.

## Data Availability Statement

The datasets generated for this study are available on request to the corresponding author.

## Author Contributions

VL-L led the project, guided the experiments, procured funding, and drafted the manuscript. FA, DP, and VL-L were involved in the experimental design, conducting the experiments, and collecting the data. All authors reviewed the manuscript.

## Conflict of Interest

VL-L is principal investigator of Contract N° 852 1 between University Paris-Sud and ProbioSwiss. The remaining authors declare that the research was conducted in the absence of any commercial or financial relationships that could be construed as a potential conflict of interest.
